# Office and 24-hour heart rate and target organ damage in hypertensive patients

**DOI:** 10.1186/1471-2261-12-19

**Published:** 2012-03-22

**Authors:** Ángel García-García, Manuel A Gómez-Marcos, José I Recio-Rodríguez, Maria C Patino-Alonso, Emiliano Rodríguez-Sánchez, Cristina Agudo-Conde, Luis García-Ortiz

**Affiliations:** 1 Primary Care Research Unit, La Alamedilla Health Center. REDIAPP. IBSAL.SACyL, Salamanca, Spain; 2 Statistics Department, University of Salamanca, Salamanca, Spain

**Keywords:** Heart rate, Hypertension, Blood pressure monitoring, ambulatory, Carotid arteries, Hypertrophy, left ventricular, Kidney disease

## Abstract

**Background:**

We investigated the association between heart rate and its variability with the parameters that assess vascular, renal and cardiac target organ damage.

**Methods:**

A cross-sectional study was performed including a consecutive sample of 360 hypertensive patients without heart rate lowering drugs (aged 56 ± 11 years, 64.2% male). Heart rate (HR) and its standard deviation (HRV) in clinical and 24-hour ambulatory monitoring were evaluated. Renal damage was assessed by glomerular filtration rate and albumin/creatinine ratio; vascular damage by carotid intima-media thickness and ankle/brachial index; and cardiac damage by the Cornell voltage-duration product and left ventricular mass index.

**Results:**

There was a positive correlation between ambulatory, but not clinical, heart rate and its standard deviation with glomerular filtration rate, and a negative correlation with carotid intima-media thickness, and night/day ratio of systolic and diastolic blood pressure. There was no correlation with albumin/creatinine ratio, ankle/brachial index, Cornell voltage-duration product or left ventricular mass index. In the multiple linear regression analysis, after adjusting for age, the association of glomerular filtration rate and intima-media thickness with ambulatory heart rate and its standard deviation was lost. According to the logistic regression analysis, the predictors of any target organ damage were age (OR = 1.034 and 1.033) and night/day systolic blood pressure ratio (OR = 1.425 and 1.512). Neither 24 HR nor 24 HRV reached statistical significance.

**Conclusions:**

High ambulatory heart rate and its variability, but not clinical HR, are associated with decreased carotid intima-media thickness and a higher glomerular filtration rate, although this is lost after adjusting for age.

**Trial Registration:**

ClinicalTrials.gov: NCT01325064

## Background

Traditionally, high heart rate (HR) at rest has been directly associated with a risk of cardiovascular (CV) disorders, both in the general and in the elderly population [1], as well as in the patients with previous diseases [2,3]. Recently, the behavior of HR in different scenarios has been assessed, with the conclusion that the beneficial decrease in HR depends on the previous pathology and CV risk factors of the patient [4]. Thus, HR and blood pressure have been shown to intervene in the development of CV complications in a synergistic manner [5]. Although several studies have found an association between HR and cardiovascular risk [6,7], which HR value may have beneficial effects remains unclear [8]. It also remains unclear whether resting HR has a greater association with cardiovascular risk. As a result, several types of measurements have been carried out to establish the prognostic value of HR, including resting HR, ECG computed HR, 24-hour mean HR, HR during sleep, or HR after an exercise test [2,9]. Several authors have found an association with microinflammatory responses [10], while others consider that low HR variability (HRV) implies greater mortality than normal variability [11,12]. It also has been found that moderate intensity exercise is sufficient to improve HRV [13]. The kidney is one of the main target organs of arterial hypertension, and a worsening of renal function is a powerful predictor of cardiovascular risk [8]. Some results have shown that heart rate is an independent predictor for the prevalence of microalbuminuria in hypertensive patients with cardiovascular risk factors [14]. In addition, proteinuria values may be increased in subjects with an elevated HR, even in normotensive individuals [15]. The association of HR (both sleep and awake HR) and its variability with vascular, renal and cardiac target organ damage (TOD) in a cohort of hypertensive patients has not been clearly established [16,17].

The aim of the present study therefore was to assess the association between office and ambulatory HR and its variability with the parameters that assess the presence of vascular, renal and cardiac target organ damage.

## Methods

### Study design and population

This was a cross-sectional study performed in a primary care setting. We included 360 hypertensive patients without heart rate lowering drugs, aged 30-80 years, and seen in their primary care clinics between January 2008 and June 2010 through consecutive sampling. All patients enrolled agreed to take part in the study. The protocol was approved by an independent ethics committee of Salamanca University Hospital (Salamanca, Spain), and all participants signed the corresponding informed consent forms.

Sample size calculation indicated that the 360 patients included in the study were sufficient to detect a minimum correlation coefficient between heart rate or its variability and subclinical organ damage parameters of 0.15 in a two-sided test, with a significance level of 0.05 and a power of 0.81.

### Blood pressure, heart rate and heart rate variability measurement

Clinical blood pressure and heart rate measurements were obtained by performing three measurements of systolic blood pressure (SBP) and diastolic blood pressure (DBP), with a validated sphygmomanometer, OMRON M7 model (Omron Health Care, Kyoto, Japan), following the recommendations of the ESH [18]. For the study, the mean of the last two measurements obtained by the nurse of the research unit was used.

Ambulatory blood pressure monitoring (ABPM) was performed on a day of standard activity with a cuff adequate for the size of the patient's arm. A control system, the Spacelabs 90207 model (Spacelabs Healthcare, Issaquah, Washington, USA), was used and validated according to the protocol of the British Hypertension Society [19]. Of the total readings, ≥ 80% were considered valid. Furthermore, for the records to be evaluable, at least 14 measurements were required during the daytime period or at least seven during the night or rest period. The monitor was scheduled to obtain blood pressure measurements every 20 minutes during the daytime and every 30 minutes during the rest period. The mean and standard deviation, as a measurement of variability of SBP, DBP, and HR of each patient, were calculated for the total 24-h, daytime and night time periods, and they were defined based on the diary reported by the patient. We considered heart rate variability (HRV) as the mean of the standard deviation of HR of each patient. Each patient completed a form specifying bedtime and wake-up time and the Spacelab was programmed to analyze the variables recorded according to the actual period of rest and activity.

### Target organ damage (TOD) evaluation

#### Cardiac assessment

The electrocardiography examination was performed with a General Electric MAC 3.500 ECG System (Niskayuna, New York, USA) that automatically measures the voltage and duration of waves and estimates the criteria of the Cornell voltage-duration product (Cornell VDP) [20] to assess LVH. LVH is defined as the voltage-duration product > 2440 mm*ms. The echocardiography examination was performed by two investigators specifically trained before the start of the study. A Sonosite Micromax device (Sonosite Inc., Bothell, Washington, USA) with a 2.5 - 3.5 MHz linear transducer was used, with the subjects lying down on their left sides. The measurements were performed according to the recommendations of the American Society of Echocardiography in mode M [21]. Left ventricular mass was calculated with the Deveroux formula corrected for the body surface to estimate the left ventricular mass index (LVMI) [22]. According to the European Hypertension Guideline of 2007, LVH was defined as an LVMI greater than 125 g/m2 in men and 110 g/m2 in women [8].

#### Renal assessment

Kidney damage was assessed by measuring the glomerular filtration rate estimated (eGFR) by the CKD-EPI (Chronic Kidney Disease Epidemiology Collaboration) equation [23] and proteinuria was assessed by the albumin/creatinine ratio following the ESH 2007 criteria [11]. Target organ damage was defined as plasma creatinine of 1.3 mg/dl or higher in men and 1.2 mg/dl or higher in women, a eGFR below 60 ml/min or albumin/creatinine ratio > 22 mg/gr in men and 31 mg/gr in women[8].

#### Assessment of carotid intima-media thickness (IMT)

Carotid ultrasonography to assess IMT was performed by two investigators trained for this before starting the study. The reliability of which was evaluated before the study began using the intraclass correlation coefficient, which showed values of 0.974 (95%CI: 0.935 - 0.990) for intra-observer agreement on repeated measurements in 20 subjects, and 0.897 (95%CI:0.740 - 0.959) for inter-observer agreement. According to the Bland-Altman analysis, the limit of inter-observer agreement was 0.022 (95%CI: -0.053 - 0.098) and the limit of intra-observer agreement was 0.012 (95%CI: -0.034 - 0.059). A Sonosite Micromax ultrasound device paired with a 5-10 MHz multifrequency high-resolution linear transducer with Sonocal software (Sonosite Inc., Bothell, Washington, USA) was used for performing automatic measurements of IMT for optimizing reproducibility. Measurements were taken from the common carotid artery after the examination of a 10-mm longitudinal section at 1 cm away from the bifurcation. We performed measurements of the anterior or proximal walls and of the posterior or distal walls in the lateral, anterior and posterior projections, following an axis perpendicular to the artery to discriminate two lines, one for the intima-blood interface and the other for the media-adventitious interface. Six measurements were obtained of the right carotid and another six of the left carotid, using mean values (mean IMT) and maximum values (maximum IMT) calculated by the software automatically. The measurements were obtained following the recommendations of the Manheim Carotid Intima-Media Thickness Consensus [24]. Mean IMT was considered abnormal if it was above 0.9 mm or if there were atherosclerotic plaques with a diameter over 1.5 mm or a focal increase of 0.5 mm or 50% of the adjacent IMT [8].

#### Evaluation of peripheral artery disease

This parameter was evaluated using the ankle-brachial index (ABI) and was performed in the morning in patients who had not consumed coffee or tobacco for at least 8 hours prior to measurement in an ambient temperature of 22-24°C. With the feet uncovered and the patient in a supine position after 20 min of rest, the pressure in the upper and lower extremities was measured using a portable Minidop Es-100Vx Doppler system (Hadeco, Inc., Arima, Miyamae-ku, Kawasaki, Japan). The probe was applied at posterior tibial artery at an angle of approximately 60° relative to the direction of blood flow. The transducer's cuff was quickly inflated on each ankle to about 30 mm Hg above the systolic pressure, and the pressure was then allowed to descend (by about 2 mmHg per second) until the first sound corresponding to the systolic pressure was heard. Blood pressure was also measured in both arms (measured twice at 3-5 minute intervals). The ABI was calculated separately for each foot by dividing the higher of the two systolic pressures in the ankle by the higher of the two systolic pressures in the arm. Target organ damage was considered present if the ABI was lower than 0.9 [25] and missing value if ABI ≥ 1.30.

### Statistical analysis

Continuous variables were expressed as the mean ± standard deviation (SD), while qualitative variables were expressed on the basis of their frequency distribution. The Pearson correlation coefficient was used to estimate the relationship between quantitative variables. The multivariate analysis involved eighteen multiple linear regression models with mean IMT (nine models) and CKD-EPI (nine models) as dependent variables. We included as independent variables, clinical HR, 24-hour HR, awake HR, sleep HR, 24-hour HRV, awake HRV, sleep HRV, N/D HR ratio and N/D HRV ratio, one in each model, and age as the adjusted variable. Logistic regression analysis was performed, including as dependent variable the absence "0" or presence "1" of any TOD and as independent variables, using the enter method, age, gender, antihypertensive drugs, systolic N/D (night/day) ratio, office systolic blood pressure, waist circumference, atherogenic index (total cholesterol/HDL-cholesterol), smoking, diabetes mellitus and 24-hour HR in the first model and 24-hour HRV in the second model. An α risk of 0.05 was established as the limit of statistical significance. The SPSS/PC+ version 15.0 (SPSS Inc., Chicago, Illinois, USA) statistical package was used throughout.

## Results

Table 1 shows the general characteristics of the patients, cardiovascular risk factors, blood pressure, HR and its variability (HRV) measured with different methods, as well as the cardiac (18.3%), vascular (23.2%), renal (18.0%) and overall TOD (45.0%). The patients on antihypertensive drugs were the 44.7% (161) and the 89% of these were on one or two drugs. The most commonly prescribed being angiotensin receptor antagonists (34%) and diuretics (33%) followed by ACE inhibitors (24%) and dihydropyridine calcium antagonists (7%). The diabetics patients on drug therapy were 51 (68.9%). The 50% were on metformin, 30% on sulfonylurea and 17% on insulin.

**Table 1:** General demographic and clinics characteristics

Variables		Total (360)
Age		56 ± 11
Gender (male %)		231 (64.2%)
Smoking n (%)		89 (24.7%)
Diabetes Mellitus n (%)		74 (21.0%)
Cardiovascular disease n (%)		18 (5.0%)
BMI		28.1 ± 3.9
Waist circumference (cm)		97.6 ± 11.2
Antihypertensive drugs n (%)		161 (44.7%)
Diabetics patients on drug therapy n (%)		51 (68.9%)

Office BP (mmHg)	SBP	138 ± 16
	DBP	87 ± 10
	HR (bpm)	74 ± 12
ABPM 24 h (mmHg)	SBP	123 ± 11
	DBP	76 ± 8
	HR (bpm)	72 ± 9
	HRV (SDHR)	10.7 ± 3.3
Awake ABPM (mmHg)	SBP	127 ± 11
	DBP	79 ± 9
	HR (bpm)	76 ± 10
	HRV (SDHR)	9.6 ± 3.3
Sleep ABPM (mmHg)	SBP	112 ± 14
	DBP	66 ± 9
	HR (bpm)	62 ± 8
	HRV (SDHR)	6.8 ± 3.4

Systolic night/day ratio		0.89 ± 0.08
Diastolic night/day ratio		0.84 ± 0.10
Night/day HR		0.82 ± 0.07
Night/day HRV		0.76 ± 0.38
Fasting glucose (mg/dl)		99 ± 30
Creatinine (mg/dl)		0.92 ± 0.20
Total Cholesterol (mg/dl)		209 ± 37
Triglycerides (mg/dl)		126 ± 78
Atherogenic index (Total Cholesterol/HDL-Cholesterol)		4.11 ± 1.13

Renal	Albumin/Creatinine (mg/gr)	18 ± 79
	eGFR (ml/min/1.73 m^2^)	86 ± 15
Heart	Cornell VDP (mmxms)	1482 ± 780
	LVMI (g/m^2^)	108 ± 33
Vascular	IMT mean (mm)	0.74 ± 0.12
	left ABI	1.08 ± 0.16
	right ABI	1.09 ± 0.15
Renal TOD, n (%)		58 (18.0%)
Heart TOD, n (%)		65 (18.3%)
Vascular TOD, n (%)		82 (23.2%)
Some TOD, n (%)		162 (45.0%)

Data for qualitative variables are expressed as n (%) and quantitative variables as mean ± standard deviation. The duration of hypertension is indicated in years. BMI: body mass index; SBP: Systolic blood pressure; DBP: Diastolic blood pressure; HR: Heart rate; HRV: Heart rate variability; SDHR:Heart rate standard deviation; VDP: Voltaje-duration product; IMT: Intima-media thickness; LVMI: Left ventricular mass index; ABI: ankle/brachial index. eGFR: estimate glomerular filtration rate, TOD: Target organ damage, bpm: beats per minute

Tables 2 shows the correlation between clinical and ambulatory HR and HRV and the measurements of the parameters used to assess the presence of TOD. Age showed a negative correlation with ambulatory HR and HRV, but not clinical HR, for 24-hour, awake, and sleep HR. GFR estimated with the CKD-EPI equation showed a positive correlation with ambulatory HR (24-hour, awake, and sleep) and HRV (sleep), whereas the albumin/creatinine ratio did not reach statistical significance. Mean IMT showed a negative correlation with the ambulatory HR and 24-hour, awake, and sleep HRV (only HRV). We found no correlation between any of the measurements of HR and HRV with respect to ABI, Cornell VDP or LVMI measured by echocardiography. Finally, the night/day ratio (both systolic and diastolic) showed a negative correlation with all HR and HRV measurements, except for sleep HR and N/D HRV ratio.

**Table 2:** Correlations between HR and HRV and parameters that assess target organ damage

	Age	Album/creatinine index	eGFR	Mean IMT	ABI	Cornell VDP	LVIM g/m2	Systolic night/day ratio	Diastolic night/day ratio
Office HR	-0.089	0.007	0.084	-0.053	0.001	-0.102	-0.104	-0.175†	-0.190†
24 h HR	-0.228†	0.059	0.196†	-0.150†	-0.100	-0.021	0.050	-0.133*	-0.159†
Awake HR	-0.222†	0.048	0.191†	-0.147†	-0.094	-0.028	0.051	-0.162†	-0.188†
Sleep HR	-0.136*	0.095	0.141†	-0.053	-0.101	-0.010	0.054	0.012	0.020
24 h HRV	-0.248†	-0.094	0.095	-0.232†	-0.052	-0.075	0.012	-0.187†	-0.230†
Awake HRV	-0.222†	-0.088	0.071	-0.212†	-0.047	-0.075	0.014	-0.095	-0.146†
Sleep HRV	-0.292†	-0.073	0.132*	-0.252†	-0.013	-0.045	0.050	-0.147†	-0.131*
N/D ratio HR	0.115*	0.058	-0.071	0.130*	-0.014	0.038	-0.002	0.267†	0.318†
N/D ratio HRV	-0.161†	-0.033	0.095	-0.107*	0.024	0.005	0.045	-0.092	-0.031
**Correlations between HR and HRV and parameters that assess target organ damage (No diabetics)**									

Office HR	-0,038	-0,029	0,039	-0,040	0,014	-0,077	-0,160	-0,174†	-0,205†
24 h HR	-0,174†	-0,011	0,152*	-0,101	-0,085	0,052	0,070	-0,157†	-0,201†
Awake HR	-0,168†	-0,018	0,140*	-0,095	-0,082	0,037	0,064	-0,192†	-0,232†
Sleep HR	-0,089	0,012	0,111	-0,016	-0,097	0,080	0,078	-0,004	-0,005
24 h HRV	-0,205†	-0,072	0,056	-0,184†	-0,047	-0,062	-0,001	-0,194†	-0,245†
Awake HRV	-0,188†	-0,071	0,045	-0,166†	-0,047	-0,049	0,004	-0,089	-0,151*
Sleep HRV	-0,234†	-0,053	0,111	-0,222†	0,044	-0,005	0,079	-0,167†	-0,152*
N/D ratio HR	0,100	0,037	-0,043	0,103	-0,024	0,066	0,007	0,277†	0,335†
N/D ratio HRV	-0,113	-0,023	0,080	-0,089	0,081	0,032	0,071	-0,125*	-0,049
**Correlations between HR and HRV and parameters that assess target organ damage (Diabetics)**									

Office HR	-0,299†	0,041	0,247*	-0,110	-0,146	-0,173	0,173	-0,182	-0,139
24 h HR	-0,519†	0,122	0,345†	-0,394†	-0,151	-0,218	-0,024	-0,092	-0,049
Awake HR	-0,523†	0,098	0,358†	-0,406†	-0,160	-0,208	0,010	-0,109	-0,075
Sleep HR	-0,393†	0,182	0,239*	-0,245†	-0,119	-0,245*	-0,025	0,035	0,082
24 h HRV	-0,455†	-0,159	0,248*	-0,444†	-0,087	-0,119	0,073	-0,163	-0,174
Awake HRV	-0,376†	-0,145	0,181	-0,402†	-0,072	-0,150	0,066	-0,110	-0,122
Sleep HRV	-0,482†	-0,102	0,257*	-0,297*	-0,087	-0,140	-0,268	0,004	0,005
N/D ratio HR	0,233*	0,127	-0,194	0,292*	0,072	-0,033	-0,048	0,249*	0,265*
N/D ratio HRV	-0,276*	-0,025	0,185	-0,068	-0,074	-0,035	-0,214	0,123	0,099

Office HR: Office Heart rate; 24 h HR: 24 hours Heart rate; Awake HR: Awake Heart rate; Sleep HR: Sleep Heart rate; 24 h HRV: 24 hours heart rate variability; Awake HRV: Awake heart rate variability; Sleep HRV: Sleep heart rate variability; N/D ratio HR: night/day heart rate ratio; eGFR: estimate glomerular filtration rate; IMT: Intima-media Thickness; ABI: ankle/brachial index; VDP: Voltage-duration product; LVMI: left ventricular mass index; * p < 0.05 † p < 0.01

These correlations were stronger in diabetics patients and the subgroup without antihypertensive treatments and weaker in the subgroup with antihypertensive drugs, losing statistical significance in some of the correlations of HR and HRV with IMT and systolic and diastolic blood pressure night/day ratio. The statistics signification of the association found between HR and HRV with IMT and eFGR was lost after adjusting for age.

**Figure 1:**
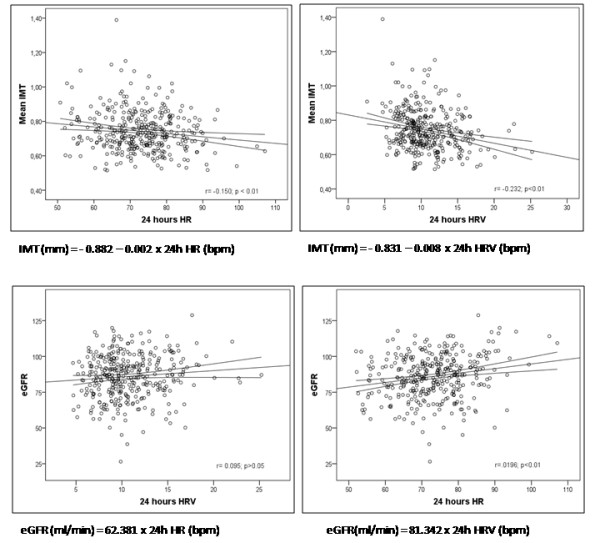
**Simple linear regression of IMT and eGFR with 24-hour HR and 24-hour HRV**. eGFR: estimate glomerular filtration rate; IMT: Intima-media thickness; HR: Heart rate; HRV: Heart rate variability; bpm: beat per minute.

Figure 1 shows the simple linear regression of IMT and eGFR as dependent variables, and 24-hour HR and 24-hour HRV as independent variables. Of note is the observation that for each 10-bpm increment in HR, the IMT value decreases by 0.02 mm and the eGFR increases by 3.24 ml/min/1.73 m^2^.

According to the logistic regression analysis (Table 3), the predictors of damage to any target organ were age (Odds Ratio (OR) = 1.034 and OR = 1.033) and night/day systolic blood pressure ratio (OR = 1.425 and OR = 1.512). Neither 24 HR nor 24 HRV reached statistical significance.

**Table 3:** Logistic regression analysis

Model 1				
**Variable:**	**B**	**Sig**.	**OR**	**OR (95% IC)**

24 hours HR	-0.005	0.691	0.995	0.968 to 1.022
Age	0.033	0.005	1.034	1.010 to 1.058
Gender	0.414	0.121	1.512	0.897 to 2.549
Antihypertensive drugs (1)	0.052	0.832	1.054	0.650 to 1.709
Night/Day SBP ratio*10	0.354	0.015	1.425	1.071 to 1.896
Smoking (1)	-0.357	0.196	0.699	0.407 to 1.203
Diabetes mellitus (1)	-0.493	0.089	0.611	0.346 to 1.079
Waist circumference	0.023	0.059	1.023	0.999 to 1.047
Atherogenic index	0.149	0.183	1.161	0.932 to 1.445
Office SBP	0.009	0.238	1.009	0.994 to 1.023
Constant	-8.392	< 0.001	0.000	

**Model 2**				

24 hours HRV	0.047	0.259	1.048	0.966 to 1.137
Age	0.033	0.005	1.033	1.010 to 1.057
Gender	0.402	0.130	1.495	0.888 to 2.518
Antihypertensive drugs (1)	0.059	0.812	1.061	0.653 to 1.721
Night/Day SBP ratio*10	0.413	0.009	1.512	1.108 to 2.063
Smoking (1)	-0.279	0.311	0.756	0.441 to 1.298
Diabetes mellitus (1)	-0.458	0.111	0.632	0.360 to 1.111
Waist circumference	0.021	0.070	1.022	0.998 to 1.045
Atherogenic index	0.147	0.191	1.158	0.930 to 1.442
Office SBP	0.005	0.505	1.005	0.990 to 1.021
Constant	-9.383	< 0.001	0.000	

Dependent variable: some target organ damage (heart, vascular or renal)

Independent variables: Heart rate (HR) (model 1) and its variability (HRV) (model 2) and age, gender, antihypertensive drugs, Night/Day SBP (systolic blood pressure) ratio, waist circumference, smoking, atherogenic index diabetes mellitus, Gender: 1 male, 2 female, Antihypertensive drugs, smoking, diabetes mellitus: 1 Yes. 0 No and office SBP (systolic blood pressure). OR: Odds ratio

## Discussion

The data obtained in our study suggest that there is an association between ambulatory, but not clinical, HR and its variability with respect to IMT and eGFR, whereas no association was observed with respect to albumin/creatinine index, Cornell VDP, LVMI or ABI in patients without heart rate lowering drugs. However, this association is lost after adjusting for age. On the other hand, the variable with the greatest capacity to predict the presence or absence of target organ damage was night/day systolic ratio, without HR or HRV reaching statistical significance in the logistic regression models.

The association found with ambulatory HR but not with office HR could be due to white coat phenomenon that increases and modify the basal HR in the office.

The association between HR and its variability with respect to the development of target organ damage or vascular conditions has not been clearly established. Barrios et al. [26] noted that the absence of a decrease in HR during the resting period is independently related to mortality from any cause. In a recent metaanalysis, Bangalore and Messerli et al. [4,27] found that in contrast to patients with myocardial infarction and heart failure, a beta-blocker-associated reduction in HR increased the risk of cardiovascular events and death among hypertensive patients. However, a review of prospective studies [28] in which 6928 patients were subjected to 24-hour ABPM (not treated with beta-blocking agents), with a follow-up of more than 9 years, and analyzing morbidity-mortality according to HR, concluded that in the general population HR predicts total mortality not caused by cardiovascular conditions (OR: 1.15 and 1.18, respectively).

Fácila et al. [17] studied a sample of 566 hypertensive patients, assessing HR by ABPM during activity and resting periods, and analyzing the association with the presence of TOD. The prevalence of TOD was found to be 12.4%, versus 46.7% in our study. The difference in HR between awake and sleep (10 bpm) was the same as in our study, but these authors found no association of HR with TOD in the bivariate analysis. However, in the logistic regression analysis they found a nighttime HR of over 65 bpm to be associated with an increase in TOD (OR: 2.41). Probably the higher prevalence of TOD found in this study, as well as the analysis of HR with different types of measurements, together with a higher percentage of males and patients without treatment influenced the differences found between the two studies. However, Cuspidi et al. [16] found that 48-h ambulatory HR was not associated with markers of target organ damage in the early phases of essential hypertension.

Gottsäter et al. [29] found a negative association in diabetic patients between HR, its variability and IMT. Likewise, Gautier et al. [30], in a cohort of healthy patients and patients with vascular risk factors, found a low heart rate to be related to a higher IMT. Thus, all published data follow the same line and suggest that the lower HR, assessed in different scenarios and in different groups of patients, is associate with the higher IMT. However, the results of Cuspidi et al. [16], similar to ours, in which that association disappears after adjusting for sociodemographic variables, makes it necessary to interpret these findings with caution. Nevertheless, this association was also found by Huikuri et al. [31] between HR, HR variability, and progression of focal atherosclerosis and they think it may be explained by hemodynamic factors, effects of the autonomic nervous system, or a combination of these factors. Likewise, a dysautonomic nervous system has been described as a possible explanation for the association between HRV alteration and the progression of carotid atherosclerosis [29].

Brotman et al. [32] noted an association between high HR and a greater incidence of chronic and terminal renal disease. They attributed this to autonomic dysfunction. However, we found a positive association between HR and improved glomerular filtration, but not with microalbuminury, though the prognostic value of this observation is not clear, since the association was lost after adjustment for age. Cuspidi et al. also found no association between heart rate and renal organ damage [16]. Therefore, the association of HR and renal function assessed by GFR has not been clarified to date.

Martini et al. [33] found a low HRV to be an independent risk factor for mortality among the general population and among patients who have suffered acute myocardial infarction.

They also concluded that there is a continuous negative association between left ventricular mass and HRV, while Cuspidi et al. [16] found similar results with HR, but was not confirmed by the multivariate analysis. In this study, with patients without heart rate lowering drugs, we did not find this association.

Finally, in our study the most important predictive variable of damage to any target organ was the night/day systolic ratio (OR = 1.418). A fact that had already been suggested in 1997 by Mancia et al. [34], indicating that the most promising index from ABPM seems to be arterial pressure variability - exhibiting an independent association with target organ damage in hypertensive patients.

The main limitation of this study is its cross-sectional design, which precludes longitudinal analysis between HR, HRV and TOD. Another limitation is the selection of the study population, since sampling was performed consecutively with pragmatic and broad inclusion criteria; thus, the study population included hypertensive patients, some with diabetes and hyperlipidemia, and many patients receiving drug therapy (not HR lowering drugs). This could modify blood pressure levels and thus limit the validity of some results. Consequently, the heterogeneity of the sample could lead to some limitations when interpreting the results, though it is quite similar to the distribution of the general population of hypertensive patients with some cardiovascular risk factors.

## Conclusion

In conclusion, high ambulatory HR and its variability, but not clinical HR, were associated with a decreased carotid intima-media thickness and a higher glomerular filtration rate, although it was lost when adjusted for age. We found no association of HR with albumin/creatinine ratio, Cornell VDP, left ventricular mass index or ankle/brachial index. In summary, the data from this study indicate that the associations found between HR and its variability with the parameters that assess target organ damage is mediated principally by age. This would be contrary to consider the HR as an independent risk factor for the appearance of TOD.

However, given the discrepancies found in the literature, we consider that further prospective studies are needed in order to determine the association between HR and its variability with cardiovascular risk in the early stages of hypertension and prior to the development of TOD.

## Competing interests

The authors declare that they have no competing interests.

## Authors' contributions

AGG and LGO devised the study, designed the protocol, participated in fund raising, and prepared the draft manuscript. CPA collaborated in protocol design and data interpretation. JIRR, ERS and CAC participated in study design, data collection, and manuscript review. MAGM participated in protocol design, fund raising, analysis of results, and final review of manuscript. Finally, all authors reviewed and approved the final version of the manuscript.

## Pre-publication history

The pre-publication history for this paper can be accessed here:

http://www.biomedcentral.com/1471-2261/12/19/prepub
